# p53 Hypersensitivity Is the Predominant Mechanism of the Unique Responsiveness of Testicular Germ Cell Tumor (TGCT) Cells to Cisplatin

**DOI:** 10.1371/journal.pone.0019198

**Published:** 2011-04-21

**Authors:** Matthias Gutekunst, Moshe Oren, Andrea Weilbacher, Michael A. Dengler, Christiane Markwardt, Jürgen Thomale, Walter E. Aulitzky, Heiko van der Kuip

**Affiliations:** 1 Dr Margarete-Fischer-Bosch Institute of Clinical Pharmacology and University of Tuebingen, Stuttgart, Germany; 2 Department of Molecular Cell Biology, Weizmann Institute of Science, Rehovot, Israel; 3 Institute for Cell Biology, University of Duisburg-Essen Medical School, Essen, Germany; 4 2^nd^ Department of Internal Medicine, Robert-Bosch-Hospital, Stuttgart, Germany; University of Illinois at Chicago, United States of America

## Abstract

Consistent with the excellent clinical results in testicular germ cell tumors (TGCT), most cell lines derived from this cancer show an exquisite sensitivity to Cisplatin. It is well accepted that the high susceptibility of TGCT cells to apoptosis plays a central role in this hypersensitive phenotype. The role of the tumor suppressor p53 in this response, however, remains controversial. Here we show that siRNA-mediated silencing of p53 is sufficient to completely abrogate hypersensitivity not only to Cisplatin but also to non-genotoxic inducers of p53 such as the Mdm2 antagonist Nutlin-3 and the proteasome inhibitor Bortezomib. The close relationship between p53 protein levels and induction of apoptosis is lost upon short-term differentiation, indicating that this predominant pro-apoptotic function of p53 is unique in pluripotent embryonal carcinoma (EC) cells. RNA interference experiments as well as microarray analysis demonstrated a central role of the pro-apoptotic p53 target gene NOXA in the p53-dependent apoptotic response of these cells. In conclusion, our data indicate that the hypersensitivity of TGCT cells is a result of their unique sensitivity to p53 activation. Furthermore, in the very specific cellular context of germ cell-derived pluripotent EC cells, p53 function appears to be limited to induction of apoptosis.

## Introduction

TGCT develop from pre-malignant intratubular germ cell neoplasias and can be histologically classified into seminomas and non-seminomas. Seminomas consist of sheets of cells with clear cytoplasm and are relatively homogenous. Non-seminomas include yolk sac tumors and choriocarcinomas with extraembryonic cell differentiation, teratomas with somatic cell differentiation, and EC [1]. Dependent on the histological type, non-seminomas are composed of a disorganized mixture of differentiated somatic cell types and extraembryonic cells, together with EC cells. EC cells represent the pluripotent stem cell compartment in these tumors and retain their capability for self-renewal as well as differentiation into multiple cell types. In contrast to most other solid malignancies, TGCT can be cured at a rate in excess of 80% by Cisplatin-based chemotherapy regimens even in advanced metastasized phases [2], [3]. These unique response rates have been linked to an intrinsic hypersensitivity to DNA damaging agents, as observed in several human EC lines derived from TGCT [4], [5]. Various attempts have been made to understand the molecular mechanisms behind this hypersensitivity, mostly by comparing Cisplatin-sensitive TGCT cell lines with Cisplatin-resistant clones established from the same origin by continuous treatment with increasing doses of Cisplatin. Mechanisms involved in Cisplatin resistance include reduced drug uptake, increased drug efflux and increased intracellular detoxification [6], [7]. Resistance has also been attributed to an enhanced DNA repair capacity [1]. Cell lines from TGCT have been shown to express relatively low amounts of the Xeroderma Pigmentosum group A (XPA) protein, which has been linked to hypersensitivity as a result of a reduced Nucleotide Excision Repair (NER) capacity [8]–[10]. Exogenous expression of XPA in sensitive TGCT cells, however, did not reduce sensitivity to Cisplatin in these cells [11]. The unique sensitivity of TGCT cells to Cisplatin has also been linked to an extensive and rapid induction of apoptosis and to a reduced ability to induce cell cycle arrest, probably caused by altered functionality of the p53 pathway in these cells [12]. In contrast to many other solid tumors, most TGCTs not only harbor wild type (wt) p53 but also express this tumor suppressor protein in higher than normal levels [13], [14]. The presence of wtp53 overexpression in many TGCT has been proposed as an important biological explanation for their chemo-sensitivity [15]. However, studies on the importance of p53 function for Cisplatin hypersensitivity have yielded conflicting results: whereas several in vitro and in vivo studies have suggested a central role of p53 in the hypersensitivity of TGCT and embryonic stem cells to Cisplatin [16]–[18], others failed to confirm such a role [19], [20].

In the present study, we demonstrate a close relationship between p53 protein levels and the extent of apoptosis in pluripotent TGCT cells. Interestingly, this hypersensitivity to the pro-apoptotic function of p53 was not limited to DNA damage-inducing agents, but could also be detected when p53 was stabilized in a non-genotoxic manner.

## Results

### Hypersensitivity of EC cells to Cisplatin is p53-dependent

**Figure 1:**
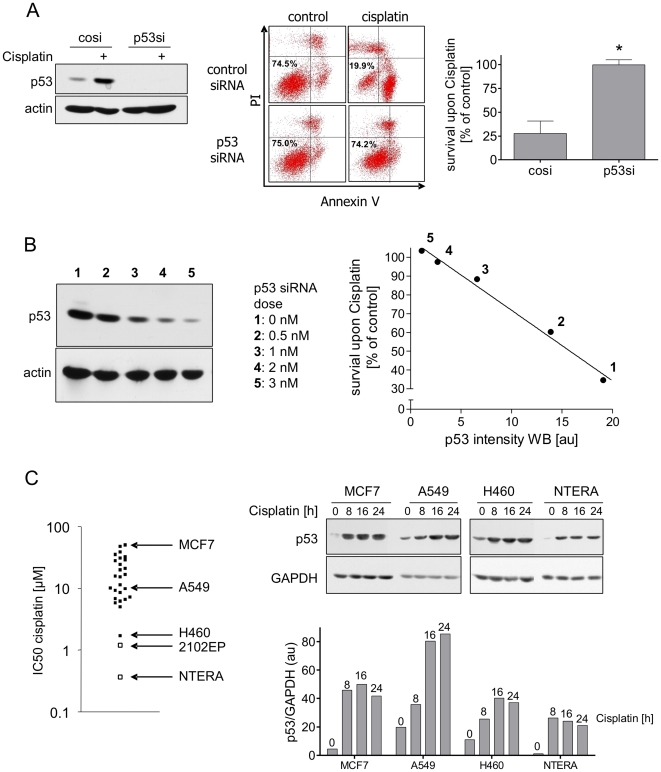
Hypersensitivity of NTERA-2D1 is entirely dependent on p53. (A) siRNA-mediated knockdown of p53 rescues NTERA-2D1 from Cisplatin-induced apoptosis. Cells were transfected with siRNA and incubated for 48 h prior to Cisplatin treatment (16 h). Left panel: verification of p53 knockdown by Western Blot analysis. Middle and right panel: cells were stained with Annexin-V/FITC and PI and analyzed by flow cytometry. Graph reflects means ±SD of survival after Cisplatin relating to corresponding controls from 5 experiments (*P* = 0.0003). (B) Cisplatin-induced apoptosis correlates with the amount of p53 protein (densitometric values). Cells were transfected with indicated amounts of p53-targeting siRNA 48 h before Cisplatin treatment (16 h) and analyzed by Western Blot. (C) Hypersensitivity of NTERA-2D1 is not due to extraordinary high levels of p53. Left panel: Cells were treated with indicated concentrations of Cisplatin to determine IC_50_ in a panel of 28 cell lines (the two TGCT cell lines NTERA-2D1 and 2102EP are indicated by open squares) using MTT assay. Three cell lines (MCF-7, A549, H460) with differing Cisplatin sensitivity were chosen to compare p53 protein levels with NTERA-2D1. Right panel: Cells were treated with Cisplatin for indicated times and whole protein lysates were then applied to Western Blot analysis. Graph reflects intensity of p53 bands determined by densitometry relating to the amount of GAPDH.

Most cell lines derived from EC undergo apoptosis upon exposure to very low concentrations of Cisplatin. We first analyzed whether p53 is essential for Cisplatin-induced apoptosis. To address this question, we used RNA interference (RNAi) to specifically knockdown p53 expression. As shown in Figure 1A (left panel), treatment of NTERA-2D1 cells with p53 siRNA led to a complete loss of both p53 protein expression and accumulation upon Cisplatin. Importantly, this RNAi-mediated loss of p53 accumulation was sufficient to completely rescue NTERA-2D1 cells from Cisplatin-induced apoptosis (Figure 1A, middle and right panels). The same result was observed for the EC cell line 2102EP (Figure S1). In addition, in TGCT cells we found a tight p53 siRNA dose response relationship for the rescue from Cisplatin-induced apoptosis, demonstrating that in these cells the amount of p53 protein is pivotal for their sensitivity to Cisplatin (Figure 1B).

It has been demonstrated that most TGCTs are characterized by constitutively high levels of p53 [21], [22]. To evaluate if extraordinarily high p53 levels are responsible for the hypersensitivity we compared p53 protein levels in four wtp53 cell lines (MCF7, A549, H460, and NTERA-2D1 cells) selected from a panel of 28 cell lines characterized by different sensitivities to Cisplatin (Figure 1C, left panel). Neither constitutive p53 protein levels nor p53 accumulation upon Cisplatin differed significantly among these cell lines (Figure 1C, right panel). In fact, the two most resistant cell lines, A549 and MCF7, accumulated even more p53 upon Cisplatin treatment as compared to the sensitive cell lines (Figure 1C, right panel). Therefore, hypersensitivity of NTERA-2D1 cells to Cisplatin does not appear to be due to exceptionally high levels of p53. This suggests that the specific cellular context of pluripotent EC cells is what dictates the predominantly pro-apoptotic function of p53 in these cells.

### The PI3 family kinases ATM, ATR, and DNA-PK are dispensable for p53-dependent induction of apoptosis upon Cisplatin

**Figure 2:**
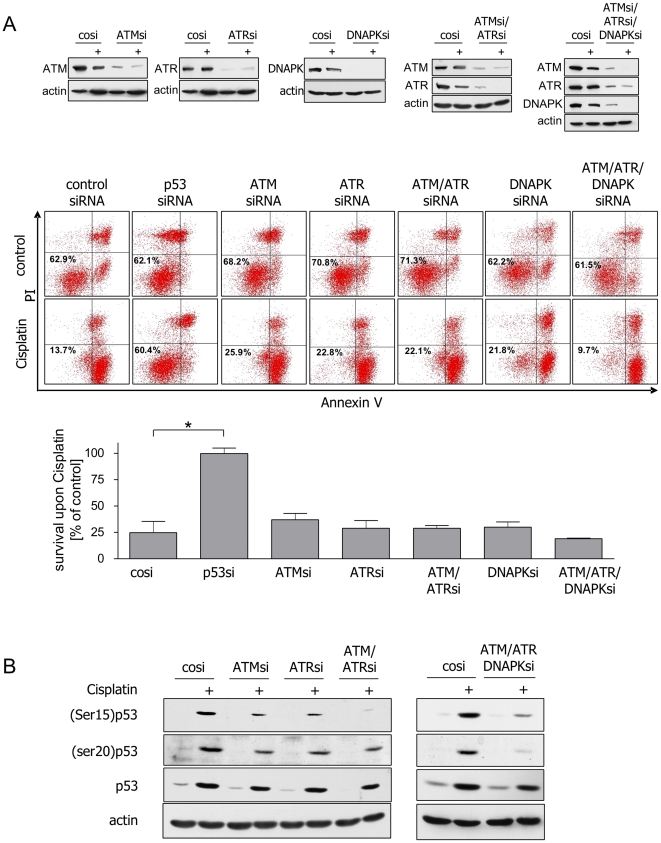
ATM, ATR and DNA-PK are dispensable for Cisplatin-induced apoptosis. (A) Prominent upstream kinases of p53 are dispensable for Cisplatin hypersensitivity of NTERA-2D1 cells. Cells were transfected with siRNA and incubated for 48 h prior to Cisplatin treatment (16 h). Upper panel: verification of different knockdowns by Western Blot. Middle and lower panel: cells were stained with Annexin-V/FITC and PI and analyzed by flow cytometry. Graph reflects means ±SD of survival after Cisplatin relating to corresponding controls (cells treated with the same siRNA but cultivated in absence of Cisplatin) from 3 experiments. (B) Phosphorylation on serines 15 and 20 does not seem to be a requirement for p53 accumulation in NTERA-2D1 cells upon Cisplatin. Knockdowns described in (A) cultivated in presence or absence of Cisplatin were analyzed for posttranslational modifications and amount of p53 protein by Western Blot.

We next asked whether DNA damage signaling pathways are responsible for the preferential activation of the pro-apoptotic functions of p53 in NTERA-2D1 cells. The major upstream transducers of DNA damage signaling include ATM, ATR, and DNA-PK [23]. We therefore examined the roles of these protein kinases by RNAi experiments. All siRNAs efficiently repressed their target genes and blocked protein expression (Figure 2A, upper panel). Surprisingly, neither knockdown of ATM, ATR, or DNA-PK alone nor ATM/ATR double knockdown or ATM/ATR/DNA-PK triple knockdown had any significant protective effect on survival of NTERA-2D1 cells upon Cisplatin. Rather, triple knockdown of ATM, ATR, and DNA-PK even increased Cisplatin-induced apoptosis (Figure 2A).

Phosphorylation of serine residues near the N terminus of p53 (in particular serine 15 and 20) by members of the PI3-kinase family and the CHK family has been reported to be critical for stabilization of p53 upon DNA damage [24], [25]. As expected, phosphorylation on serine 15 was partially dependent on ATM and ATR and disappeared almost completely upon ATM/ATR double or ATM/ATR/DNA-PK triple knockdown (Figure 2B). p53 phosphorylation on serine 20 was partially affected by ATM, ATR, or ATM/ATR double knockdown. Triple knockdown of ATM, ATR, and DNA-PK effectively reduced serine 20 phosphorylation (Figure 2B). Surprisingly, however, we observed a significant p53 accumulation despite effective knockdown of these upstream PI3-kinases (Figure 2B). These results indicate that phosphorylation of p53 on serines 15 and 20 may not be required for the pro-apoptotic activity of p53 in NTERA-2D1 cells upon Cisplatin.

### Hypersensitivity to Cisplatin depends on concomitant induction of PUMA and NOXA

**Figure 3:**
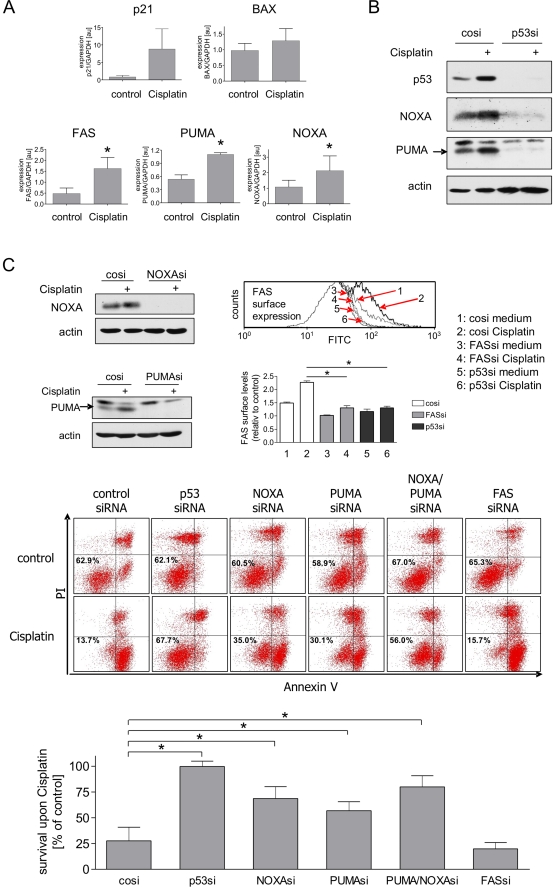
NOXA and PUMA mediate Cisplatin-induced apoptosis downstream of p53. (A) Cisplatin induces p21, FAS, NOXA and PUMA. Cells were treated with Cisplatin for 8 h. RNA was extracted and transcribed to cDNA. Expression of selected p53 target genes was determined by PCR. Graphs reflect means ±SD from 3 experiments of mRNA levels normalized to GAPDH (*: *P*<0.05). (B) Constitutive and Cisplatin induced NOXA and PUMA protein levels depend on p53. Cells were transfected with control siRNA or p53 siRNA and incubated for 48 h prior to Cisplatin treatment (16 h). Efficacy of p53 silencing as well as expression of PUMA and NOXA protein were then analyzed by Western Blot. (C) NOXA and PUMA are important mediators of Cisplatin-induced apoptosis. Cells were transfected with siRNA and incubated for 48 h prior to Cisplatin treatment (16 h). Upper panel: validation of siRNA efficacy by Western Blot (for NOXA and PUMA; upper left panels) or by evaluation of surface expression using a CD95/FAS specific antibody conjugated to FITC and flow cytometry (for FAS; upper right panel; *: *P*<0.05). Lower panel: cell survival upon Cisplatin treatment. Cells were stained with Annexin-V/FITC and PI and analyzed by flow cytometry. Graph reflects means ±SD of survival after Cisplatin relating to corresponding controls from 3 experiments (*: *P*<0.05).

To analyze which targets are responsible for the predominant pro-apoptotic function of p53 in NTERA-2D1 cells, we studied the expression of a panel of pro-apoptotic and cell cycle regulatory p53 targets. Cisplatin induced the transcription of p21, FAS/CD95, PUMA, and NOXA, whereas induction of BAX was only minimal (Figure 3A). Importantly, p21 transcript levels were found to be extremely low in NTERA-2D1 cells when compared to normal fibroblasts or other cell lines and did not lead to production of detectable p21 protein (Figure S2). PUMA, NOXA, and FAS expression was completely dependent on p53 since p53 silencing led to an almost complete loss of those factors both constitutively as well as upon Cisplatin treatment (Figure 3B and Figure 3C, upper right panel). To test whether PUMA, NOXA, or FAS play a role in the hypersensitive phenotype of NTERA-2D1 cells, we pre-treated these cells with the corresponding siRNAs. All siRNAs efficiently repressed their target genes in NTERA-2D1 (Figure 3C, upper panel) and 2102EP cells (Figure S3, upper panel). Knockdown of PUMA, and to an even greater extent NOXA, significantly inhibited Cisplatin-induced death of NTERA-2D1 (Figure 3C, middle and lower panels) and 2102EP cells (Figure S3, middle and lower panels). Silencing both PUMA and NOXA almost completely blocked the induction of apoptosis in NTERA-2D1 cells (Figure 3C, middle and lower panels). In contrast, FAS knockdown did not change the sensitivity of NTERA-2D1 (Figure 3C) or 2102EP cells (Figure S3, middle and lower panels) to Cisplatin. These data indicate that PUMA and NOXA are the major effectors of p53 in these cells, and p53-mediated transactivation of both genes is critical for the hypersensitive phenotype.

### NTERA-2D1 cells are hypersensitive to p53 induction independent of DNA damaging agents

**Figure 4:**
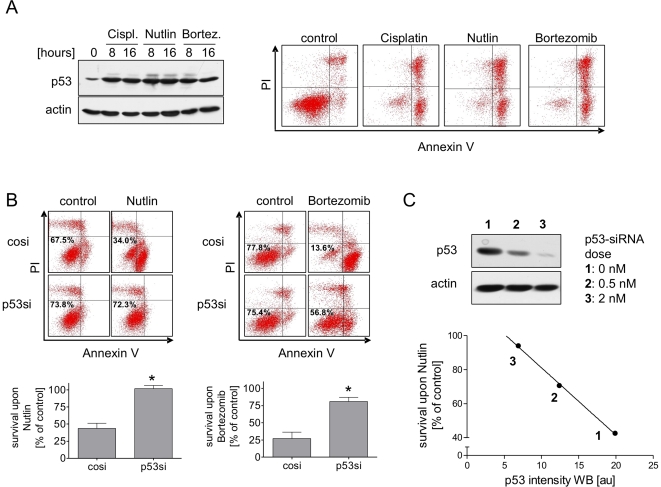
NTERA-2D1 cells are hypersensitive to pro-apoptotic functions of p53 independent of DNA damage. (A) Hypersensitivity of NTERA-2D1 cells is also observed upon DNA damage-independent accumulation of p53. Cells were treated with Cisplatin, Nutlin-3, or Bortezomib, respectively for indicated times and p53 protein was analyzed by Western Blot (left panel). In addition, cells treated for 24 h were stained with Annexin-V/FITC and PI and analyzed by flow cytometry (right panel). (B) Apoptosis upon the non-genotoxic agents Nutlin-3 and Bortezomib is dependent on p53. Cells were transfected with siRNA and cultivated for 48 h prior to Nutlin-3 or Bortezomib treatment (16 h). Cells were stained with Annexin-V/FITC and PI and analyzed by flow cytometry. Graph reflects means ±SD of survival after Cisplatin relating to corresponding controls from 3 experiments (*P* = 0.0003 and *P* = 0.0014 for Nutlin-3 and Bortezomib, respectively). (C) Nutlin-3 induced apoptosis directly correlates with the amount of p53 protein (determined by densitometry). Cells were transfected with indicated amounts of p53-targeting siRNA 48 h before Nutlin-3 treatment and analyzed by Western Blot.

In NTERA-2D1 cells, the p53 protein content is quantitatively related to Cisplatin sensitivity (Figure 1). In addition, important DNA damage transducers such as ATM, ATR, and DNA-PK do not play a major role in Cisplatin hypersensitivity (Figure 2). We therefore asked whether DNA damage-induced p53 activation is at all a prerequisite for the hypersensitive phenotype. To that end, we studied the sensitivity of NTERA-2D1 cells to DNA damage-independent inducers of p53. It has been demonstrated that inhibition of Mdm2 function by Nutlin-3 stabilizes p53 in a non-genotoxic manner [26]. In addition, p53 protein also accumulates in the absence of DNA damaging agents upon treatment with the proteasome inhibitor Bortezomib [27]. As shown in Figure 4A (left panel), p53 accumulation in NTERA-2D1 cells upon Nutlin-3 and Bortezomib was similar to that seen after Cisplatin. The same result was also obtained for 2102EP cells (Figure S4A). In contrast to Cisplatin, neither Nutlin-3 nor Bortezomib elicited comparable phosphorylation of p53 at serine residues 15 and 20 (not shown), confirming the DNA damage-independent accumulation of p53. Importantly, both compounds led to the same rapid and extensive induction of apoptosis as observed with Cisplatin (Figure 4A; right panel). Again, apoptosis was dependent on p53, as pretreatment with p53 siRNA was sufficient to rescue those cells completely from Nutlin-3 and at least partially from Bortezomib-induced apoptosis (Figure 4B). The same result was also obtained with RITA, another inhibitor of the Mdm2-p53 interaction [28] (data not shown). Again, these data could be confirmed in a second EC cell line (2102EP; Figure S4B).

In analogy to the results obtained with Cisplatin, Nutlin-3-induced apoptosis was also correlated with the absolute levels of p53, as demonstrated by the p53 siRNA dose-response relationship in Figure 4C. These data not only confirm that TGCT cells are hypersensitive to p53 regardless of how p53 accumulation is achieved, but also imply that defects in DNA repair are not major prerequisites for hypersensitivity, since non-genotoxic Mdm2 antagonists or proteasome inhibitors are not expected to inflict substantial DNA damage. This is further supported by the demonstration that NTERA-2D1 cells are capable of removing Cisplatin-DNA adducts when apoptosis is blocked by inhibition of caspase activation (Figure S5), indicating that a rapid and massive induction of apoptosis may precede the onset of DNA repair in embryonal carcinoma cells.

### The p53-dependent hypersensitivity of NTERA-2D1 cells is lost upon short-term differentiation

**Figure 5:**
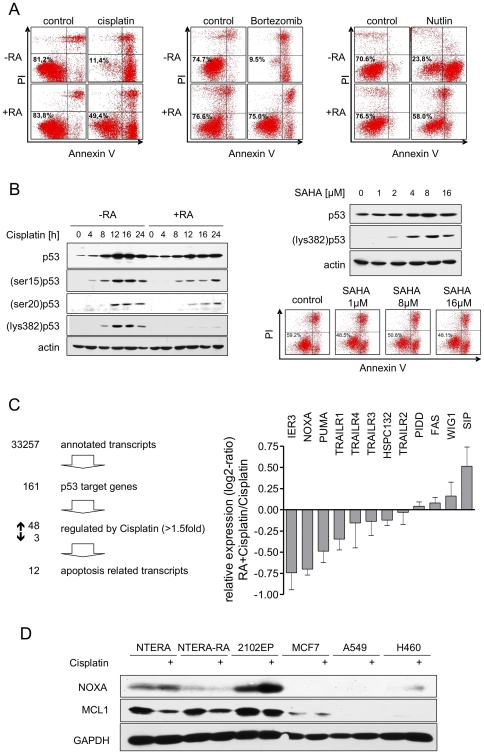
Short-term differentiation of NTERA-2D1 by RA causes a loss in hypersensitivity. Cells were partially differentiated with RA for 48 h prior to drug treatment. (A) Short-term differentiation rescues NTERA-2D1 from cell death induced by DNA damaging and DNA damaging independent agents. Cells were treated with Cisplatin, Nutlin-3 or Bortezomib, respectively for 16 h, stained with Annexin-V/FITC and PI and analyzed by flow cytometry. (B) Posttranslational modifications seem to play a minor role in loss of hypersensitivity upon short-term differentiation. Left panel: differentiated and control cells were treated with Cisplatin for indicated times and analyzed by Western Blot. Right panel: upon differentiation with RA, cells were pre-incubated with indicated concentrations of histone deacetylase inhibitor SAHA for 2 h followed by Cisplatin treatment. Cells were stained with Annexin-V/FITC and PI and analyzed by flow cytometry and protein levels of p53 and acetylation on lysine 382 were analyzed by Western Blot. (C) Expression level of NOXA upon Cisplatin is reduced in NTERA-2D1 cells pre-treated with RA. Differentiated and control cells were treated with Cisplatin for 6 h, RNA was isolated and applied to microarray analysis to assess global gene expression. Left panel: selection criteria for Cisplatin-induced p53 target genes related to apoptosis. Right panel: expression levels of the 12 apoptosis-related p53 targets upon Cisplatin were compared to cells differentiated prior to Cisplatin treatment. Graph reflects means ±SD from 3 experiments. (D) NOXA basal and Cisplatin-induced protein levels are higher in non-differentiated NTERA-2D1 and 2102EP cells than those of differentiated NTERA-2D1 (NTERA-RA) or other tumor cell lines. Cells were treated with Cisplatin for 16 h and harvested. Lysates were then used for Western Blot analysis.

Pluripotent EC cell lines derived from TGCT such as NTERA-2D1 can be induced to undergo terminal differentiation along a neuronal lineage by all-trans-retinoic acid (RA) [29]. We asked whether the hypersensitivity to p53 is unique to the pluripotent non-differentiated state or is maintained also upon differentiation. Indeed, NTERA-2D1 cells lose their hypersensitivity to Cisplatin, Bortezomib, and Nutlin-3 already after 48 h of RA treatment (Figure 5A). Because the sensitivity of the non-differentiated cells depends on p53, we asked whether this predominant pro-apoptotic p53 response is altered after short-term differentiation. Notably, pre-treatment of NTERA-2D1 cells with RA did not reduce constitutive p53 protein levels and had only a minor effect on p53 protein accumulation upon Cisplatin (Figure 5B, left panel). We therefore sought to determine whether the changes in p53 function might have been triggered by differences in posttranslational modifications. As shown in Figure 5B (left panel), Cisplatin-induced phosphorylation of p53 on serines 15 and 20 was detectable in both undifferentiated and short-term differentiated NTERA-2D1 cells, even though it was slightly attenuated in cells pre-treated with RA. Interestingly, however, Cisplatin-induced acetylation on lysine 382 was almost completely abrogated after short-term differentiation (Figure 5B, left panel). To evaluate whether this reduced acetylation might be the cause for the observed differential sensitivity to Cisplatin, we pre-treated cells cultivated in the presence of RA with the histone deacetylase inhibitor SAHA. Although at concentrations above 2 µM SAHA completely rescued cisplatin-induced p53 acetylation on lysine 382, it failed to render RA-treated cells hypersensitive to Cisplatin (Figure 5B, right panel). Hence, differential acetylation on lysine 382 does not seem to play a major role in the reduced sensitivity of short-term differentiated NTERA-2D1 cells.

To examine changes in p53-regulated gene expression upon Cisplatin after RA treatment, we performed gene expression microarray analysis. Out of a total of 161 transcripts defined as p53-responsive by previously described criteria [30], [31], 51 were changed at least 1.5 fold upon Cisplatin treatment of undifferentiated cells (Figure 5C, left panel; Table S1). Gene ontology analysis identified 12 of these as apoptosis-related. We compared these apoptosis-related transcripts in both undifferentiated (control) and differentiated (RA treated) cells. Interestingly, besides IER3, NOXA and PUMA were suggested to undergo the most pronounced reduction upon pre-treatment with RA (Figure 5C, right panel). Importantly, pre-treatment of NTERA-2D1 cells with RA not only reduced Cisplatin-induced NOXA but also constitutive NOXA protein levels whereas Mcl-1 remained unchanged upon RA (Figure 5D). Furthermore, relative to the three other wtp53 tumor cell lines analyzed in this study, the non-differentiated NTERA-2D1 as well as 2102EP cells showed extraordinary high levels of both constitutive and Cisplatin-induced NOXA (Figure 5D). These results indicate that NOXA might play an important role in Cisplatin hypersensitivity of TGCT cells.

## Discussion

Pluripotent TGCT cells are extremely sensitive to apoptotic stimuli, explaining the excellent clinical results in treatment of these tumors with Cisplatin-containing drug regimens. Therefore, understanding the mechanisms underlying this hypersensitivity may open the road to manipulate other tumor types in order to enhance the therapeutic index of cytotoxic therapy. Analysis of various potential mechanisms, including altered drug transport, detoxification mechanisms and DNA repair [32], did not unequivocally identify the critical cause of the extraordinary sensitivity of TGCT cells. We found that p53 is required for the hypersensitive phenotype in these cells: (i) silencing of p53 abrogates hypersensitivity in cell lines derived from TGCTs, (ii) the absolute level of p53 protein upon Cisplatin treatment determines the extent of apoptosis in these cells, and iii) the hypersensitive biological response to p53 is not limited to DNA damage-induced activation of this tumor suppressor protein. Furthermore, our data demonstrate for the first time that in these cells the p53 targets NOXA and PUMA are important effectors of this predominant pro-apoptotic function whereas the p53 upstream kinases ATM, ATR, and DNA-PK are dispensable.

A pivotal role of p53 in TGCT hypersensitivity has been implicated many years ago. Thus, it was noted that TGCTs rarely contain p53 mutations [13], [14], but TGCT patients who did not respond to chemotherapy could be linked to tumors with mutated p53 [33]. In addition, high levels of wtp53 protein were detected by immunohistochemistry in TGCT [21], and constitutively high levels of p53 were also detected in many cell lines derived from TGCT [22], [34]. Lutzker et al. found that, in isogenic mouse teratocarinoma cells, a higher rate of DNA damage-induced apoptosis correlated with higher basal levels of exogenous p53 [16]. Therefore, it has been postulated that high p53 protein content with latent transcriptional activity may be the prerequisite for the rapid activation of p53 and the concomitant loss of viability following DNA damage in these cells. Our data demonstrate that hypersensitivity depends on induction of p53 but does not necessarily require extraordinary high levels. By comparing p53 among different tumor cell lines we found that both constitutive as well as Cisplatin-induced p53 levels were actually even lower in the most sensitive cell line, NTERA-2D1, when compared to resistant cell lines. These results are in line with findings from Kersemaekers et al., who compared p53 levels of seminomas and non-seminomas with normal testis as well as breast and colon cancer cell lines, and found relatively low amounts of p53 in TGCT cells [19].

Whereas most investigators came to the conclusion that p53 is required for the extreme sensitive phenotype of TGCT cells, some studies have failed to support a predominant role of p53. Burger et al. compared NTERA-2D1 cells (harboring wtp53) with NCCIT cells (mtp53) and found no significant difference in sensitivity to Cisplatin between these cell lines [35]. However, different cell lines not only differ in their p53 status but also in many other factors important for induction of DNA damage-induced apoptosis, making it difficult to draw any conclusion basing on such univariate analysis. For example, it has been demonstrated recently that defective ATM or CHK2 signaling renders p53-deficient tumor cells hypersensitive to Cisplatin, whereas the same defects had the opposite effect in tumor cells harboring wtp53 [36]. More direct evidence that p53 may be dispensable for sensitivity to Cisplatin in TGCT cells came from Burger et al.: partial inactivation of p53 by HPV16-E6 in NTERA-2D1 cells did not render these cells more sensitive to Cisplatin [20]. HPV16-E6, however, may also alter other DNA damage response pathways independent of its ability to target p53 for degradation, including the WNT pathway [37]. Using very specific RNAi-mediated silencing of p53, we and others [17] found that inhibition of p53 accumulation dramatically reduces Cisplatin sensitivity. Moreover, our data for the first time demonstrate a linear correlation between the amounts of apoptosis upon Cisplatin with the absolute level of accumulated p53.

p53 accumulation upon DNA damage is regulated by posttranslational modifications at the N terminus of the protein, in particular phosphorylation on serines 15 and 20, mediated principally by the DNA damage transducers ATM, ATR, and DNA-PK [24], [25]. Interestingly, RNAi-mediated downregulation of ATM, ATR, and DNA-PK did not prevent Cisplatin-induced p53 accumulation and subsequent induction of rapid and massive apoptosis in NTERA-2D1 cells. These data demonstrate that neither activation of these PI3K family members nor phosphorylation of p53 on serines 15 and 20 are required for hypersensitivity to Cisplatin in these cells. This is in line with our findings that the high sensitivity to p53-dependent apoptosis in pluripotent TGCT cells is not necessarily dependent on DNA damage. Thus, we found a comparable hypersensitivity in cells treated with non-genotoxic p53 inducers such as inhibitors of the Mdm2-p53 interaction. In analogy to the results obtained with Cisplatin, Nutlin-3-induced apoptosis in NTERA-2D1 cells was also highly related to the absolute levels of accumulated p53 protein. In concordance with these results, two recently published studies showed a high sensitivity of TGCT cells to Nutlin-3 [38], [39]. In addition, both TGCT cell lines tested in our study responded to the proteasome inhibitor Bortezomib with p53 accumulation and concomitant rapid induction of apoptosis. The role of p53 in proteasome inhibitor-induced cell death is far more controversial. E.g. it has been demonstrated that Bortezomib induces apoptosis in lymphoma and melanoma cells via stabilization of NOXA protein independently of p53 [40], [41]. However, the RNAi experiments performed in the present study clearly demonstrate an important role of p53 for Bortezomib sensitivity in TGCT cells. Although cell death could not be completely blocked, p53 silencing significantly reduced Bortezomib-induced apoptosis in both cell lines tested. These data also support previous reports on the role of p53 for proteasome inhibition-induced cell death [42], [43].

Together, these data indicate that in embryonal TGCT cells elevation of p53 protein levels is sufficient to induce apoptosis, regardless of how p53 accumulation is achieved. Therefore, therapy regimens including non-genotoxic Mdm2 or proteasome inhibitors may be as effective as cytotoxic therapies for treatment of TGCT with wtp53 status, thus avoiding the relatively marked side effects of Cisplatin such as nephrotoxicity and neurotoxicity [44], [45].

These findings also suggest that DNA repair deficiency may not be a prerequisite for hypersensitivity in these cells since DNA damage is not expected to be substantially induced by non-genotoxic agents. The view that defects in the DNA repair machinery are not critical prerequisites for hypersensitivity is further supported by our finding that NTERA-2D1 cells are capable of removing Cisplatin adducts from the DNA when apoptosis is transiently blocked by caspase inhibition (supplementary Figure S2). Therefore, the reduced repair capacity observed in TGCT cells may be related to an extremely rapid and effective apoptosis preceding the onset of DNA repair.

The exact molecular mechanisms of how p53 distinguishes between pro-apoptotic and cell cycle regulating target genes still remain unclear. The function of p53 and its promoter binding capacity is regulated by a number of posttranslational modifications but also by various cofactors [46], [47] and is therefore largely dependent on the cellular context. Our data indicate that in the particular cellular background of EC cells p53 seems to act primarily as a pro-apoptotic factor. This is in line with the view that in TGCT p53 might predominantly transactivate pro-apoptotic targets whereas the products of cell cycle regulatory genes such as p21 are almost completely absent upon Cisplatin [21], [48], [49]. In accordance with previous findings [17], our results show that p53 is able to transactivate p21 in TGCT cells upon Cisplatin. However, the absolute p21 transcript levels of the treated cells were far below those of normal control cells and did not lead to a detectable induction of p21 protein. Interestingly, the opposite was true for the pro-apoptotic BH3-only protein NOXA. We found that both constitutive and Cisplatin-induced NOXA expression is significantly higher in NTERA-2D1 and 2102EP cells compared to other cell lines. The predominant role of NOXA in the sensitivity of EC cells to Cisplatin is demonstrated by the observation that silencing of NOXA rendered those cells significantly more resistant. The same effect, albeit less pronounced, was seen for PUMA. In our TGCT model, combined silencing of NOXA and PUMA had additive effects and almost completely rescued NTERA-2D1 cells from Cisplatin-induced apoptosis, indicating that the pro-apoptotic function of p53 is predominantly mediated by induction of NOXA and PUMA. Interestingly, a synergistic induction of apoptosis by NOXA and PUMA has also been reported for HeLa and MEF cells [50]. In contrast to NOXA and PUMA, silencing of FAS/CD95 had no effect on Cisplatin-induced apoptosis in NTERA-2D1 cells, despite an effective induction of its expression, indicating that the CD95 apoptotic pathway is not relevant for hypersensitivity to Cisplatin in these cells.

NTERA-2D1 are highly malignant EC cells, which have retained their pluripotent stem cell properties [51] but can be induced to differentiate and thereby lose their tumorigenicity by treatment with RA [29]. We found that hypersensitivity to Cisplatin in NTERA-2D1 cells is lost already upon short-term treatment with RA. Therefore, this cell model seems to perfectly reflect the *in vivo* situation, since differentiation and loss of embryonic features as observed in mature teratomas is also accompanied by reduced chemosensitivity [1], [52]. Our results suggest that an altered p53 activity may directly be involved in this loss of sensitivity upon differentiation. This is supported by the finding that differentiation not only protected NTERA-2D1 cells from Cisplatin but also from apoptosis mediated by the non-genotoxic p53 inducers Nutlin-3, RITA, and Bortezomib. In addition, microarray analysis uncovered Cisplatin-induced p53 target genes differentially expressed in cells pre-treated with RA. Interestingly, one of the most pronounced differences was observed for NOXA, which was significantly reduced upon treatment with RA. This indicates again a prominent role of this BH3-only protein in the hypersensitivity of pluripotent TGCT cells.

In sum, our data indicate that hypersensitivity of TGCT is a result of their unique sensitivity to the pro-apoptotic effects of p53, which is lost upon differentiation. Furthermore, in this very specific cellular context of pluripotent germ cell-derived EC cells, p53 function appears to be limited to induction of apoptosis.

## Materials and Methods

### Cell culture

MCF-7, A549, and H460 cells were obtained from the NCI-60 cell panel. 2102EP cells were procured from the American Type Culture Collection (ATCC). NTERA cells were obtained from LGC Standards, Germany. Cells were cultivated in RPMI-1640 (Biochrom, Germany) with 10% FCS and glutamine. For short-term differentiation NTERA cells were treated with 10 µM retinoic acid (Sigma, Germany) for 48 h prior to experimental procedures.

### Reagents

Cisplatin was used at a concentration of 10 µM. Generally, cells were pre-incubated with inhibitors for 2 h prior to Cisplatin treatment to ensure complete target inhibition at this time point. zVADfmk (Bachem, Germany) was used at 50 µM. Nutlin-3 (Sigma, Germany) was used at 10 µM. Bortezomib (Research Chemicals, Canada) was used at 10 nM. SAHA (Biomol, Germany) was used at indicated concentrations.

### Protein expression

For total lysates the cellular pellet was resuspended in Laemmli, boiled, and sonicated. Low molecular weight protein NOXA was detected by Western blotting according to Schaegger and von Jagow [53]. Western blot was performed using following antibodies: anti-ATR, anti-p53 (Santa Cruz, USA), anti-ATM, anti-DNAPK, anti-(Lys382)p53, anti-(Ser15)p53, anti-(Ser20)p53 (Cell Signaling, USA), anti-NOXA (Calbiochem, USA), anti-PUMA (Abcam, UK), anti-p21 (BD Pharmingen, USA), anti-GAPDH (Biodesign, USA); anti-β-actin (Sigma, Germany). FAS was detected by FACS analysis using FITC conjugated anti-FAS antibody (NatuTec, Germany).

### mRNA Expression

Total RNA was extracted and cDNA prepared according to standard protocols. Expression analysis was done using equal amounts of cDNA and primer pairs as described in table 1. The relative amount of synthesized cDNA was determined through RT-qPCR (7500 Fast Real-Time PCR System or 7900HT Fast Real-Time PCR System, Applied Biosystems, USA).

**Table 1:** Primer pairs used for RT-qPCR.

gene	sense	antisense
**FAS**	5′-TGGACCCTCCTACCTCTGGTTCT-3′	5′-GCAGGGCACGCAGTCTGGTT-3′
**GAPDH**	5′-AGCCTCAAGATCATCAGCAATG-3′	5′-CACGATACCAAAGTTGTCATGGAT-3′
**NOXA**	5′-GCAGAGCTGGAAGTCGAGTGT-3′	5′-AAGTTTCTGCCGGAAGTTCAG-3′
**p21**	5′-GGCAGACCAGCATGACAGATT-3′	5′-GCGGATTAGGGCTTCCTCTT-3′
**PUMA**	5′-ACGACCTCAACGCACAGTACG-3′	5′-TCCCATGATGAGATTGTACAGGAC-3′
**BAX**	5′-ACCAAGAAGCTGAGCGAGTGT-3′	5′-ACAAACATGGTCACGGTCTGC-3′

### MTT assay

IC_50_ in different cell lines upon cisplatin was determined by MTT assay as described previously [54].

### Apoptosis

Apoptosis was assessed by FITC-conjugated Annexin-V staining (Pharmingen, USA) as described [55].

### siRNA experiments

For silencing we used siGenome SMARTpool siRNA (Dharmacon, UK). Sequences targeted by SMARTpool siRNAs are described in table 2. As a control we used siGenome Non-Targeting siRNA #1 (Dharmacon, UK). Cells were transfected using DharmaFECT#3 (Dharmacon, UK). 48 h after transfection cells were treated according to requirements. To evaluate the efficacy of siRNA silencing protein lysates were obtained and analyzed by Western Blot.

**Table 2:** siRNA target sequences.

gene	siRNA1	siRNA2	siRNA3	siRNA4
**ATM**	GCAAAGCCCUAGUAACAUA	GGGCAUUACGGGUGUUGAA	UCGCUUAGCAGGAGGUGUA	UGAUGAAGAGAGACGGAAU
**ATR**	GAACAACACUGCUGGUUUG	GCAACUCGCCUAACAGAUA	UCUCAGAAGUCAACCGAUU	GAAUUGUGUUGCAGAGCUU
**FAS**	UAGAUGAGAUCAAGAAUGA	GAAAGAAGCGUAUGACACA	GCUGGAGUCAUGACACUAA	GUUCAACUGCUUCGUAAUU
**NOXA**	AAACUGAACUUCCGGCAGA	AAUCUGAUAUCCAAACUCU	CUGGAAGUCGAGUGUGCUA	GCAAGAACGCUCAACCGAG
**p53**	GAGGUUGGCUCUGACUGUA	GCACAGAGGAAGAGAAUCU	GAAGAAACCACUGGAUGGA	GCUUCGAGAUGUUCCGAGA
**PUMA**	CGGACGACCUCAACGCACA	CCGAGAUGGAGCCCAAUUA	CCUGGAGGGUCCUGUACAA	GGCGGAGACAAGAGGAGCA
**DNAPK**	GCAAAGAGGUGGCAGUUAA	GAGCAUCACUUGCCUUUAA	GAUGAGAAGUCCUUAGGUA	GCAGGACCGUGCAAGGUUA

### Microarray analysis

Microarray analysis was performed by Microarray Facility Tübingen, Germany. To assess global gene expression, Human Gene 1.1 ST Array Plate (Affymetrix, USA) was used. Data was obtained from three independent experiments per treatment. In order to examine gene expression prior to induction of apoptosis, cells were treated with Cisplatin for 6 h before they were harvested and RNA was extracted. Microarray data are MIAME compliant and were deposited in the ArrayExpress database (www.ebi.ac.uk/arrayexpress) under accession number E-MTAB-410.

### Statistics

Data are expressed as standard deviation of the means (SD). Changes in paired samples were analyzed using two-sided paired *t*-Test.

## Supporting Information

Figure S1 **Hypersensitivity of 2102EP to Cisplatin is dependent on p53.** siRNA-mediated knockdown of p53 rescues 2102EP cells from Cisplatin-induced apoptosis. Cells were transfected with siRNA and incubated for 48 h prior to Cisplatin treatment (16 h). Left panel: verification of p53 knockdown by Western Blot. Middle and right panel: cells were stained with Annexin-V/FITC and PI and analyzed by flow cytometry. Graph reflects means ±SD of survival after Cisplatin relating to corresponding controls from 3 experiments (*P* = 0.0018).
(TIF)

Figure S2 **NTERA are characterized by low levels of p21 transcript and protein both constitutively as well as upon Cisplatin treatment.** (A) comparison of p21 transcript levels in NTERA-2D1 and primary fibroblasts isolated from human lung tissue cultivated in presence or absence of Cisplatin for 8 h. (B) comparison of p53 and p21 protein levels in NTERA-2D1 and lymphocytes cultivated in presence or absence of indicated time periods.
(TIF)

Figure S3 **NOXA and PUMA are important mediators of Cisplatin-induced apoptosis in 2102EP cells.** Cells were transfected with siRNA and incubated for 48 h prior to Cisplatin treatment (16 h). Upper panel: validation of siRNA efficacy by Western Blot (for NOXA and PUMA; upper left panels) or by evaluation of surface expression using a CD95/FAS specific antibody conjugated to FITC and flow cytometry (for FAS; upper right panel). Lower panel: cell survival upon Cisplatin treatment. Cells were stained with Annexin-V/FITC and PI and analyzed by flow cytometry. Graph reflects means ±SD of survival after Cisplatin relating to corresponding controls from 3 experiments (*: *P*<0.05).
(TIF)

Figure S4 **2102EP cells are sensitive to pro-apoptotic functions of p53 independent of DNA damage.** (A) p53 is accumulated upon Cisplatin, Nutlin, and Bortezomib in 2102EP cells. Cells were treated with Cisplatin, Nutlin-3, or Bortezomib, respectively for 16 h and p53 protein was analyzed by Western Blot. (B) Apoptosis upon the non-genotoxic agents Nutlin-3 and Bortezomib is dependent on p53. Cells were transfected with siRNA and cultivated for 48 h prior to Nutlin-3 or Bortezomib treatment (16 h). Cells were stained with Annexin-V/FITC and PI and analyzed by flow cytometry.
(TIF)

Figure S5 **NTERA cells are capable to remove DNA-Pt adducts from DNA:** (A) zVADfmk almost completely blocks Cisplatin-induced cell death in NTERA-2D1 cells. Cells were pre-treated with zVADfmk for 2 h prior to Cisplatin. After 24 h cell death was quantified using Annexin-V/PI staining. (B) NTERA cells were pre-treated with 50 µM zVADfmk to inhibit caspase activation for 2 h and then incubated with 30 µM Cisplatin for another 2 h. Cells were then washed and incubated in fresh medium containing zVADfmk for indicated time period before harvesting. Upper panel: DNA adducts were quantified as described (Liedert et al., 2006*). Lower panel: DNA was isolated and platinum was quantified by inductively-coupled-plasma mass-spectrometry (ICP MS). *: Liedert B, Pluim D, Schellens J, Thomale J (2006). Adduct-specific monoclonal antibodies for the measurement of cisplatin-induced DNA lesions in individual cell nuclei. Nucleic Acids Res 34: e47.
(TIF)

Table S1 **p53 targets regulated by Cisplatin:** Differentiated and control cells were treated with Cisplatin for 6 h, RNA was isolated and applied to microarray analysis to assess global gene expression. Table shows p53 targets changed at least 1.5 fold upon Cisplatin treatment of undifferentiated cells.
(DOC)
