# Supplementary figures and images for: p53 Hypersensitivity Is the Predominant Mechanism of the Unique Responsiveness of Testicular Germ Cell Tumor (TGCT) Cells to Cisplatin
Source: PLoS One. 2011 Apr 21;6(4):e19198. doi: 10.1371/journal.pone.0019198 (PMC3080918; doi:10.1371/journal.pone.0019198)

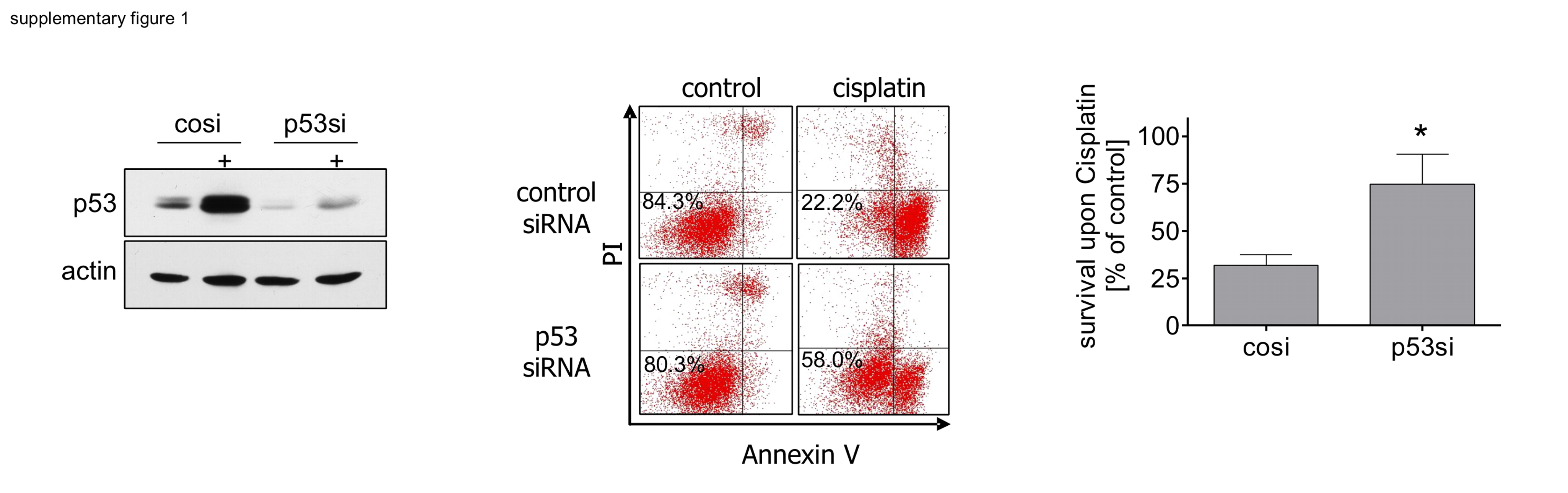

Supplement: Figure S1 — Hypersensitivity of 2102EP to Cisplatin is dependent on p53. siRNA-mediated knockdown of p53 rescues 2102EP cells from Cisplatin-induced apoptosis. Cells were transfected with siRNA and incubated for 48 h prior to Cisplatin treatment (16 h). Left panel: verification of p53 knockdown by Western Blot. Middle and right panel: cells were stained with Annexin-V/FITC and PI and analyzed by flow cytometry. Graph reflects means ±SD of survival after Cisplatin relating to corresponding controls from 3 experiments (P = 0.0018). (TIF) [file pone.0019198.s001.tif]

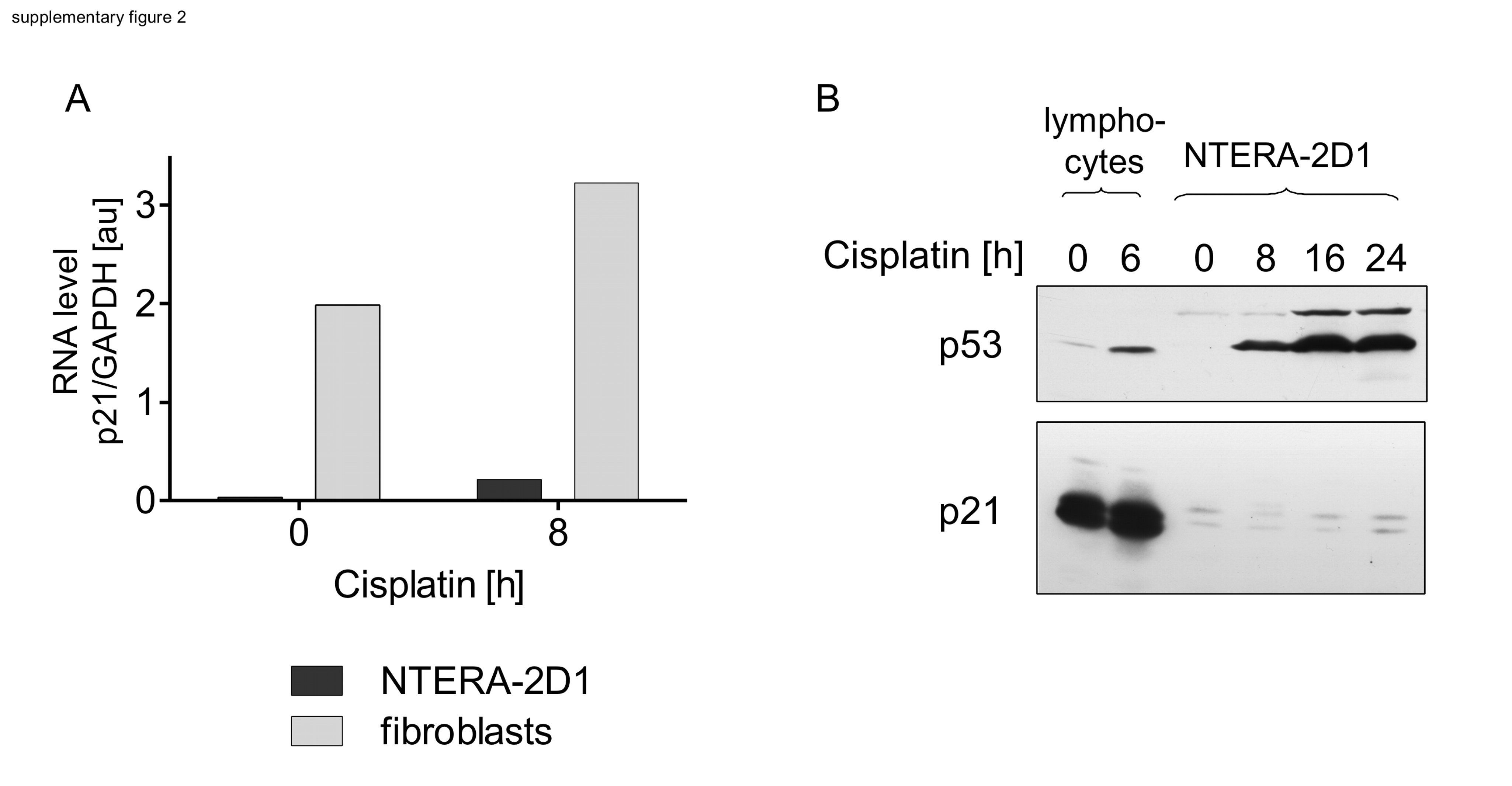

Supplement: Figure S2 — NTERA are characterized by low levels of p21 transcript and protein both constitutively as well as upon Cisplatin treatment. (A) comparison of p21 transcript levels in NTERA-2D1 and primary fibroblasts isolated from human lung tissue cultivated in presence or absence of Cisplatin for 8 h. (B) comparison of p53 and p21 protein levels in NTERA-2D1 and lymphocytes cultivated in presence or absence of indicated time periods. (TIF) [file pone.0019198.s002.tif]

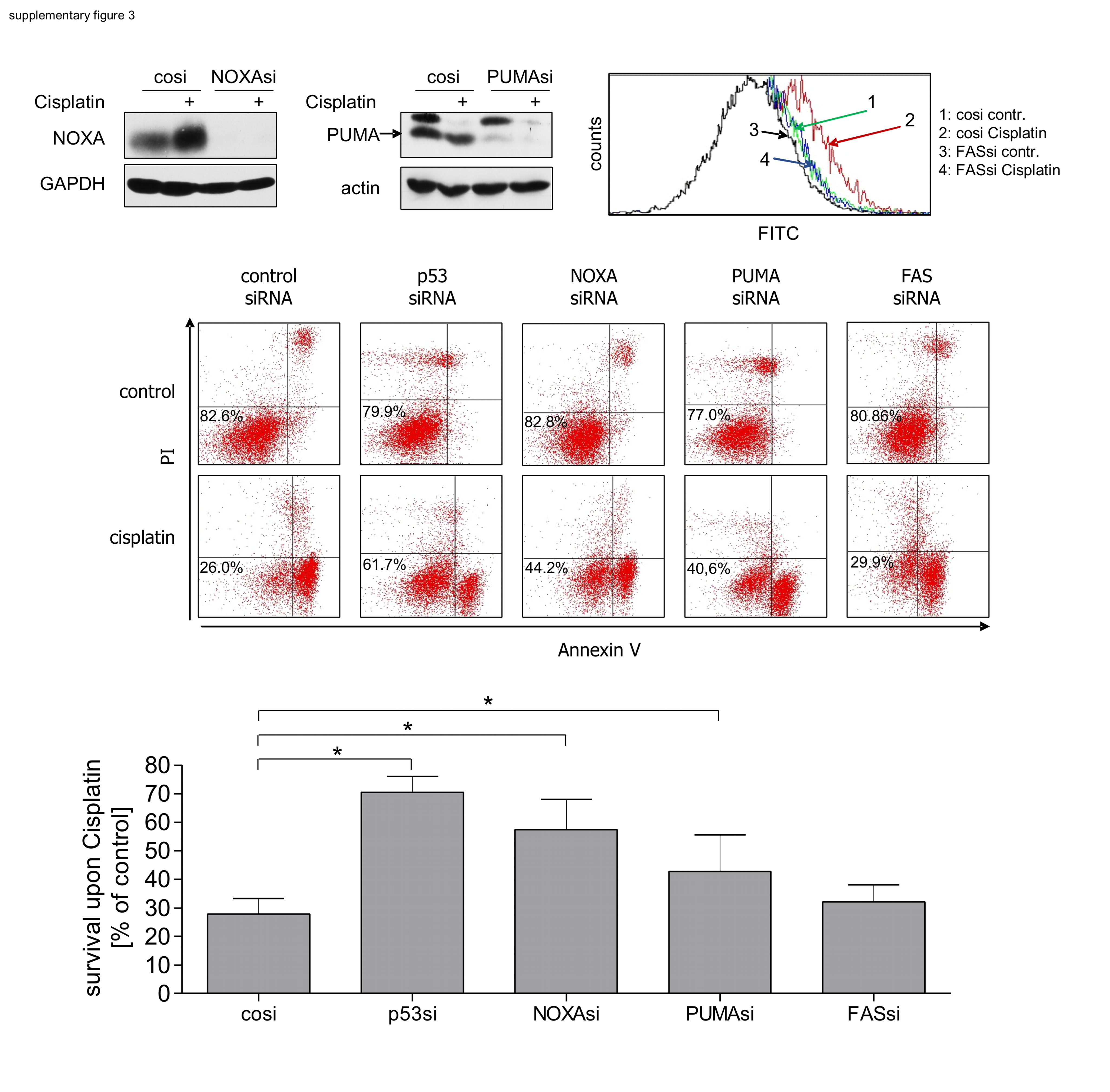

Supplement: Figure S3 — NOXA and PUMA are important mediators of Cisplatin-induced apoptosis in 2102EP cells. Cells were transfected with siRNA and incubated for 48 h prior to Cisplatin treatment (16 h). Upper panel: validation of siRNA efficacy by Western Blot (for NOXA and PUMA; upper left panels) or by evaluation of surface expression using a CD95/FAS specific antibody conjugated to FITC and flow cytometry (for FAS; upper right panel). Lower panel: cell survival upon Cisplatin treatment. Cells were stained with Annexin-V/FITC and PI and analyzed by flow cytometry. Graph reflects means ±SD of survival after Cisplatin relating to corresponding controls from 3 experiments (*: P<0.05). (TIF) [file pone.0019198.s003.tif]

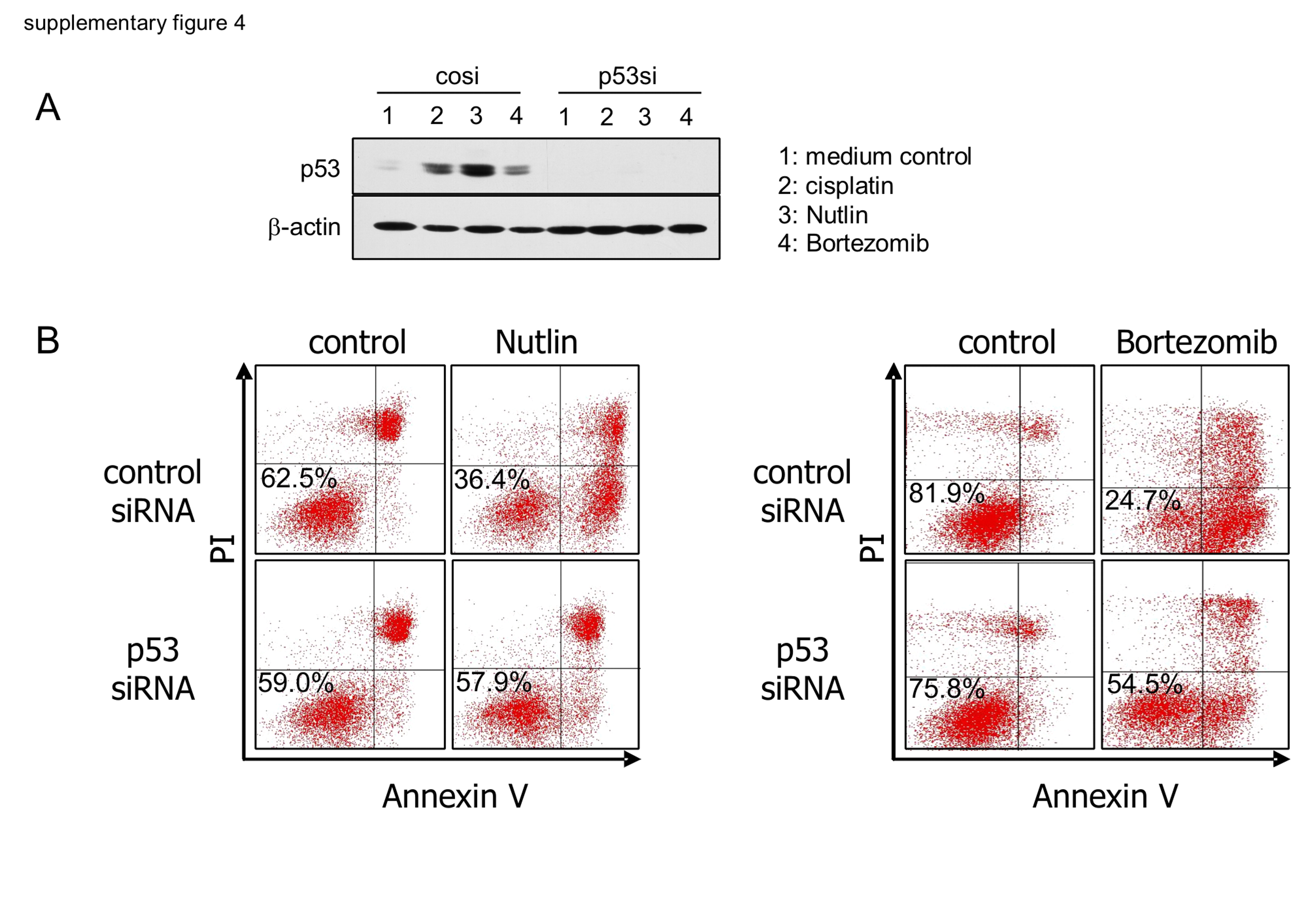

Supplement: Figure S4 — 2102EP cells are sensitive to pro-apoptotic functions of p53 independent of DNA damage. (A) p53 is accumulated upon Cisplatin, Nutlin, and Bortezomib in 2102EP cells. Cells were treated with Cisplatin, Nutlin-3, or Bortezomib, respectively for 16 h and p53 protein was analyzed by Western Blot. (B) Apoptosis upon the non-genotoxic agents Nutlin-3 and Bortezomib is dependent on p53. Cells were transfected with siRNA and cultivated for 48 h prior to Nutlin-3 or Bortezomib treatment (16 h). Cells were stained with Annexin-V/FITC and PI and analyzed by flow cytometry. (TIF) [file pone.0019198.s004.tif]

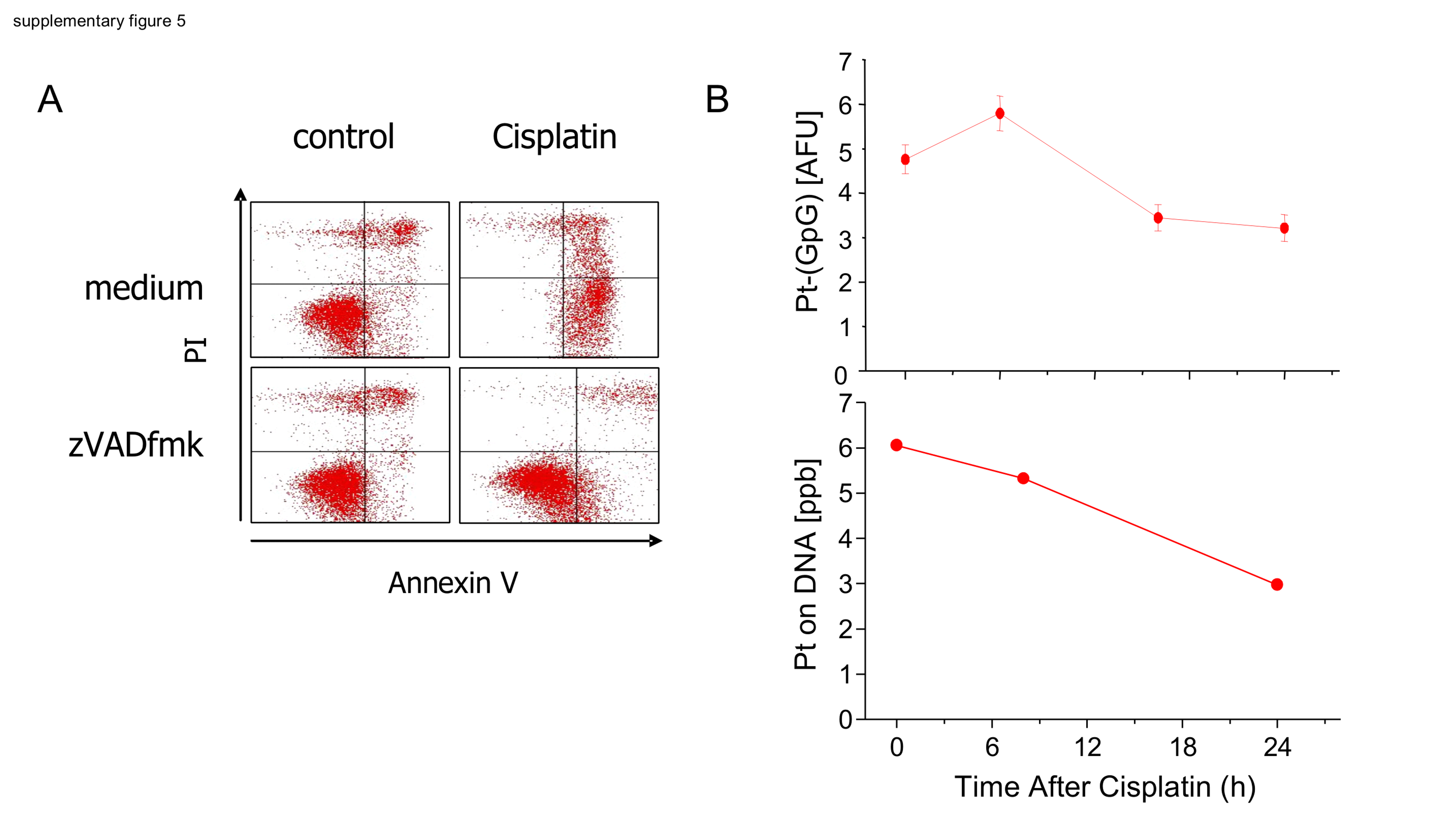

Supplement: Figure S5 — NTERA cells are capable to remove DNA-Pt adducts from DNA: (A) zVADfmk almost completely blocks Cisplatin-induced cell death in NTERA-2D1 cells. Cells were pre-treated with zVADfmk for 2 h prior to Cisplatin. After 24 h cell death was quantified using Annexin-V/PI staining. (B) NTERA cells were pre-treated with 50 µM zVADfmk to inhibit caspase activation for 2 h and then incubated with 30 µM Cisplatin for another 2 h. Cells were then washed and incubated in fresh medium containing zVADfmk for indicated time period before harvesting. Upper panel: DNA adducts were quantified as described (Liedert et al., 2006*). Lower panel: DNA was isolated and platinum was quantified by inductively-coupled-plasma mass-spectrometry (ICP MS). *: Liedert B, Pluim D, Schellens J, Thomale J (2006). Adduct-specific monoclonal antibodies for the measurement of cisplatin-induced DNA lesions in individual cell nuclei. Nucleic Acids Res 34: e47. (TIF) [file pone.0019198.s005.tif]
