# Supplementary material for: p53 Hypersensitivity Is the Predominant Mechanism of the Unique Responsiveness of Testicular Germ Cell Tumor (TGCT) Cells to Cisplatin
Source: PLoS One. 2011 Apr 21;6(4):e19198. doi: 10.1371/journal.pone.0019198 (PMC3080918; doi:10.1371/journal.pone.0019198)
Supplement: Table S1 — p53 targets regulated by Cisplatin: Differentiated and control cells were treated with Cisplatin for 6 h, RNA was isolated and applied to microarray analysis to assess global gene expression. Table shows p53 targets changed at least 1.5 fold upon Cisplatin treatment of undifferentiated cells. (DOC) [file pone.0019198.s006.doc]

**p53 targets** upregulated by Cisplatin

| **-RA: fold induction** | **+RA: fold induction** | **expression upon Cisplatin** | **Accesion number** | **Gene** | **description** | **function** |
| --- | --- | --- | --- | --- | --- | --- |
| 8,45 | 5,21 | 552,38 | NM_004864 | GDF15 | growth differentiation factor 15 | ST |
| 5,17 | 2,20 | 1061,21 | NM_006763 | BTG2 | BTG family, member 2 | R |
| 4,24 | 3,12 | 502,15 | NM_078467 | CDKN1A | cyclin-dependent kinase inhibitor 1A (p21, Cip1) | C |
| 4,10 | 3,26 | 707,64 | NM_004073 | PLK3 | polo-like kinase 3 (Drosophila) | C |
| 3,88 | 2,30 | 523,94 | NM_006622 | PLK2 | polo-like kinase 2 (Drosophila) | C |
| 3,44 | 2,10 | 223,83 | NM_033285 | TP53INP1 | tumor protein p53 inducible nuclear protein 1 | **A** |
| 3,26 | 2,49 | 120,61 | NM_019058 | DDIT4 | DNA-damage-inducible transcript 4 | A |
| 3,22 | 2,17 | 175,53 | NM_006142 | SFN | stratifin | C |
| 3,18 | 2,19 | 398,79 | NM_024417 | FDXR | ferredoxin reductase | M |
| 2,66 | 2,51 | 417,04 | NM_014454 | SESN1 | sestrin 1 | C |
| 2,61 | 2,08 | 57,56 | NM_000593 | TAP1 | transporter 1, ATP-binding cassette, sub-family B (MDR/TAP) | T |
| 2,53 | 2,15 | 530,36 | NM_031459 | SESN2 | sestrin 2 | C |
| 2,53 | 1,96 | 118,49 | NM_003844 | TNFRSF10A | tumor necrosis factor receptor superfamily, member 10a | **A** |
| 2,53 | 2,20 | 83,86 | NM_000043 | FAS | Fas (TNF receptor superfamily, member 6) | **A** |
| 2,46 | 2,32 | 574,37 | NM_003840 | TNFRSF10D | tumor necrosis factor receptor superfamily, member 10d, decoy with truncated death domain | **A** |
| 2,35 | 1,97 | 115,24 | NM_013376 | SERTAD1 | SERTA domain containing 1 | C |
| 2,30 | 2,18 | 711,11 | NM_000107 | DDB2 | damage-specific DNA binding protein 2, 48kDa | R |
| 2,19 | 1,51 | 663,38 | NM_005749 | TOB1 | transducer of ERBB2, 1 | P |
| 2,12 | 2,34 | 77,56 | NM_019099 | C1orf183 | chromosome 1 open reading frame 183 | - |
| 2,05 | 1,91 | 88,51 | NM_018494 | LRDD | leucine-rich repeats and death domain containing | A |
| 2,05 | 1,19 | 459,28 | NM_001706 | BCL6 | B-cell CLL/lymphoma 6 | TR |
| 1,99 | 2,06 | 85,42 | NM_003841 | TNFRSF10C | tumor necrosis factor receptor superfamily, member 10c, decoy without an intracellular domain | **A** |
| 1,98 | 1,69 | 244,40 | NM_001924 | GADD45A | growth arrest and DNA-damage-inducible, alpha | C |
| 1,95 | 1,43 | 72,28 | NM_001127240 | BBC3 | BCL2 binding component 3 | **A** |
| 1,91 | 1,46 | 983,39 | NM_003620 | PPM1D | protein phosphatase 1D magnesium-dependent, delta isoform | C |
| 1,87 | 0,94 | 75,43 | NM_001013398 | IGFBP3 | insulin-like growth factor binding protein 3 | P |
| 1,83 | 1,31 | 654,19 | NM_000602 | SERPINE1 | serpin peptidase inhibitor, clade E (nexin, plasminogen activator inhibitor type 1), member 1 | P |
| 1,83 | 1,39 | 123,73 | NM_001040619 | ATF3 | activating transcription factor 3 | TR |
| 1,82 | 2,06 | 43,34 | NM_002309 | LIF | leukemia inhibitory factor (cholinergic differentiation factor) | P |
| 1,78 | 0,83 | 40,71 | NM_001198 | PRDM1 | PR domain containing 1, with ZNF domain | TR |
| 1,71 | 1,78 | 121,42 | NM_015920 | RPS27L | ribosomal protein S27-like | R |
| 1,70 | 1,35 | 445,83 | NM_004417 | DUSP1 | dual specificity phosphatase 1 | C |
| 1,70 | 1,67 | 364,40 | NM_016399 | TRIAP1 | TP53 regulated inhibitor of apoptosis 1 | **A** |
| 1,68 | 1,70 | 133,14 | NM_004628 | XPC | xeroderma pigmentosum, complementation group C | R |
| 1,64 | 1,58 | 461,86 | NM_004881 | TP53I3 | tumor protein p53 inducible protein 3 | **A** |
| 1,63 | 1,37 | 26,52 | AB007455 | TP53TG1 | TP53 target 1 (non-protein coding) | SR |
| 1,62 | 1,57 | 665,80 | NM_003842 | TNFRSF10B | tumor necrosis factor receptor superfamily, member 10b | **A** |
| 1,61 | 1,61 | 306,95 | NM_007026 | DUSP14 | dual specificity phosphatase 14 | ST |
| 1,61 | 1,19 | 84,64 | NM_016545 | IER5 | immediate early response 5 | - |
| 1,58 | 1,68 | 915,27 | NM_020375 | C12orf5 | chromosome 12 open reading frame 5 | M |
| 1,57 | 1,57 | 664,03 | NM_002592 | PCNA | proliferating cell nuclear antigen | R/P |
| 1,55 | 1,88 | 1367,05 | NM_021127 | PMAIP1 | phorbol-12-myristate-13-acetate-induced protein 1 | **A** |
| 1,55 | 1,18 | 70,19 | NM_018400 | SCN3B | sodium channel, voltage-gated, type III, beta | IT |
| 1,54 | 1,73 | 1800,64 | NM_003897 | IER3 | immediate early response 3 | **A** |
| 1,54 | 1,57 | 143,99 | NM_022470 | ZMAT3 | zinc finger, matrin type 3 | **A** |
| 1,53 | 1,37 | 806,01 | NM_006253 | PRKAB1 | protein kinase, AMP-activated, beta 1 non-catalytic subunit | M/ST |
| 1,53 | 1,22 | 253,30 | NM_004148 | NINJ1 | ninjurin 1 | ADH |

**p53 targets downregulated by Cisplatin**

| **-RA: fold induction** | **+RA: fold induction** | **expression upon Cisplatin** | **Accesion number** | **Gene** | **description** | **function** |
| --- | --- | --- | --- | --- | --- | --- |
| 0,65 | 0,66 | 87,49 | NM_003550 | MAD1L1 | mitotic arrest deficient-like 1 | ST |
| 0,63 | 0,70 | 240,97 | NM_153831 | PTK2 | protein tyrosine kinase 2 | C |
| 0,47 | 0,7 | 57,15 | NM_000136 | FANCC | Fanconi anemia, complementation group C | R |

A : apoptosis

ADH : adherence

C : cell cycle regulation

R : repair

M: metabolism

T: transport

IT: ion transport

P: proliferation

TR: regulation of transcription
